# Quality, reliability, and stigmatizing content of sexual and gender minority health videos on Chinese short-form video platforms

**DOI:** 10.3389/fpubh.2026.1915092

**Published:** 2026-10-14

**Authors:** Haoyang Hu, Zekai Yu, Fei Yang

**Affiliations:** 1 Department of Neurology, The First Medical Center, Chinese PLA General Hospital, Beijing, China; 2 Medical School of Chinese PLA, Beijing, China; 3 School of Computer Science and Technology, Hangzhou Dianzi University, Hangzhou, Zhejiang, China

**Keywords:** communication framing, health misinformation, LGBTQ+ health, sexual and gender minority health, short-form video, social media, stigma

## Abstract

**Background:**

Sexual and gender minority (SGM) populations increasingly rely on digital platforms for health information, yet the quality, reliability, and stigmatizing content of SGM health videos remain unclear. This study evaluated SGM health-related short-form videos on the Chinese platforms Douyin and Bilibili.

**Methods:**

We conducted a cross-sectional content analysis of prominently ranked videos retrieved using 15 research-team-developed search terms during the prespecified data-collection period (January 1–May 1, 2026). For each search term, the first 10 results returned under each platform's default ranking were recorded, yielding 150 candidate records per platform before deduplication. Eligible videos were assessed for uploader type, content theme, engagement metrics, Global Quality Score (GQS), modified DISCERN reliability, medical accuracy, misinformation, and an exploratory stigma-to-affirmation communication score. Non-parametric tests, Cramér's V with 95% confidence intervals, and unadjusted Spearman correlations were used.

**Results:**

Of 300 candidate records, 194 videos were included (118 Douyin, 76 Bilibili). Individual users and healthcare professionals were the most common uploaders. HIV/STI prevention and mental health were the leading themes, whereas healthcare access, privacy protection, HPV vaccination, and cancer screening were underrepresented. Overall quality was moderate [median GQS = 3.0; interquartile range (IQR), 2.0–4.0], but reliability was limited (median modified DISCERN = 2.0; IQR, 1.0–3.0). High-quality and highly reliable videos accounted for 38.1% and 19.1%, respectively. Misinformation appeared in 27.8% of videos, and 17.0% contained stigmatizing, pathologizing, fear-based, or discriminatory elements. Raw cumulative engagement metrics were strongly intercorrelated but showed weak unadjusted associations with quality, reliability, accuracy, or the exploratory communication score.

**Conclusion:**

Among prominently ranked SGM health videos identified using the predefined search strategy, overall quality was moderate, reliability was limited, thematic coverage was uneven, and misinformation and stigmatizing communication remained present. Raw engagement metrics showed weak associations with quality-related scores. These findings describe a time-bounded algorithmic snapshot and should not be generalized to all content on either platform.

## 1 Introduction

Sexual and gender minority (SGM) populations, including lesbian, gay, bisexual, transgender, queer, and gender-diverse individuals, represent heterogeneous communities with diverse health needs. Accumulating evidence indicates that SGM populations experience disproportionate burdens across multiple health domains, including mental health, HIV and other sexually transmitted infections (STIs), sexual and reproductive health, preventive care, cancer screening, substance use, and access to culturally competent healthcare services (1–3). These disparities are not inherent consequences of sexual orientation or gender identity; rather, they are shaped by minority stress, social stigma, discrimination, structural inequities, limited access to affirming care, and insufficient provider training in SGM health (4–7). Accurate, evidence-based, and non-stigmatizing health communication is therefore essential for improving health literacy, supporting timely help-seeking, and reducing health inequities among SGM populations.

Health information seeking has increasingly shifted from clinical settings to digital environments. Social media platforms have become important sources of health-related information because of their accessibility, immediacy, interactivity, and broad reach (8, 9). For SGM-related health topics, online environments may be particularly influential, as individuals concerned about privacy, disclosure, stigma, or discrimination may seek information about sexual health, mental health, HIV/STI prevention, pre-exposure prophylaxis (PrEP), post-exposure prophylaxis (PEP), gender identity, or healthcare access through anonymous or semi-anonymous digital channels before engaging with formal healthcare services (10–14). Short-form video platforms provide a low-threshold medium through which users can encounter, share, and discuss SGM health information.

In China, Douyin, the Chinese version of TikTok, and Bilibili are two influential video-sharing platforms with distinct communication ecologies. Douyin is characterized by algorithm-driven short-video dissemination, rapid content circulation, and high-frequency user engagement, whereas Bilibili often contains longer videos and a stronger community-based content culture. These platforms may facilitate the dissemination of SGM health information to broad audiences, including individuals with limited access to affirming offline health education. However, the same features that enable rapid dissemination may also amplify incomplete, misleading, or stigmatizing content. Unlike peer-reviewed medical resources, short-form videos are often user-generated, variably moderated, and strongly shaped by platform algorithms and audience engagement. Prior studies of health-related short videos have reported heterogeneous information quality, limited source transparency, and weak or inconsistent associations between user engagement and content reliability (15–19). Thus, popularity metrics such as likes, comments, saves, and shares may not reliably reflect medical accuracy, reliability, or educational value.

Poor-quality SGM health information may have important consequences. Inaccurate or incomplete information about HIV/STI prevention, PrEP, PEP, testing, or safer sexual practices may delay preventive care or promote ineffective risk-reduction strategies. Oversimplified messages about mental health may discourage professional help-seeking or omit crisis-related resources. Content related to transgender and gender-diverse health may be particularly vulnerable to misinformation when it lacks clinical context or overgeneralizes medical pathways. Beyond factual accuracy, videos may pathologize SGM identities, use shame- or fear-based narratives, or reinforce stereotypes. Evaluating this communication therefore requires attention to both harmful stigma and active identity-respecting support (20–22).

Minority stress theory provided the interpretive framework for this study (4, 7). The theory distinguishes distal stressors, such as prejudice, discrimination, and stigmatizing social messages, from proximal processes, such as expectations of rejection, concealment, and internalized stigma. On short-video platforms, pathologizing, discriminatory, moralizing, or fear-based health messages may constitute distal communication exposures, whereas respectful and explicitly counter-stigmatizing messages may offer a potentially protective informational context. A content analysis cannot determine whether viewers internalize these messages or experience downstream stress; it can, however, characterize the communication environment in which such processes may occur.

Although previous studies have assessed the quality and reliability of social media videos across several medical topics (15–19), most have focused on disease-specific information. Little is known about SGM health information on short-form video platforms, particularly in non-Western digital contexts. This gap is important because SGM health is a multidimensional field involving medical accuracy, health equity, stigma reduction, privacy, healthcare access, and culturally competent communication. General video quality instruments alone may not fully capture these SGM-specific concerns.

To address this gap, we evaluated prominently ranked SGM health-related videos on Douyin and Bilibili. Guided by minority stress theory, we asked: RQ1, what uploader and thematic profiles characterize the retrieved videos, and how do these profiles differ between platforms? RQ2, to what extent do the videos contain distal-stressor cues—including stigmatizing, discriminatory, pathologizing, or fear-based framing—or communication that actively counters such cues? RQ3, how are engagement metrics associated with quality, reliability, medical accuracy, misinformation, and the exploratory stigma-to-affirmation communication score? These questions concern communication content and exposure opportunities; they do not test viewers' proximal stress processes or health outcomes.

## 2 Methods

### 2.1 Study design and data collection

This cross-sectional content analysis evaluated the quality, reliability, medical accuracy, misinformation, stigmatizing content, and user engagement of SGM health-related short-form videos on two major Chinese platforms: Douyin (the Chinese version of TikTok) and Bilibili.

We searched both platforms during the prespecified data-collection window (January 1–May 1, 2026) using 15 predefined Chinese search terms related to SGM health. The research team developed the terms *a priori* from formal terminology used in clinical and public health guidance and prior SGM health literature to cover general SGM health education, mental health, sexual health, HIV/STI prevention, PrEP/PEP, transgender and gender-diverse health, stigma, discrimination, and healthcare access. The terms were not derived from focus groups, user search logs, or a formal user-facing pilot. Consequently, colloquial or euphemistic queries used by SGM users (for example, terms analogous to “circle,” “rainbow,” or “gay”) may have retrieved additional content. The complete bilingual search-term list and sampling procedures are reported in Supplementary Table S1.

To reduce account-history personalization, all searches were performed using newly created accounts with no prior browsing history, likes, comments, saves, shares, follows, or other user interactions. Each search term was entered separately under the platform's default comprehensive/relevance ranking, and the first 10 returned videos were recorded, yielding 150 candidate records per platform before deduplication. This top-10 rule captured a time-specific algorithmic snapshot rather than all relevant videos. Douyin's recommendation-centered short-video ecology and Bilibili's more community- and subscription-oriented ecology may rank and surface content differently. Results were pooled separately by platform. Within-platform duplicates were identified using the platform-specific video ID and URL; a video retrieved by more than one term was retained once. Identical content appearing separately on both platforms was treated as a platform-specific record because platform engagement was an outcome of interest.

For each included video, we extracted platform, triggering search term(s), search rank, video title, duration, uploader type, primary content theme, and cumulative engagement metrics (likes, comments, saves/collections, and shares). Publication date, engagement-capture date, view count, and uploader follower count were recorded when displayed and available. The quota of 150 candidate records per platform was specified *a priori* to balance coverage across 15 queries with the feasibility of structured consensus coding. It standardized the retrieval rule but did not create a probability sample or equalize the platforms' underlying user populations. Accordingly, the study characterizes prominently ranked content under the specified searches and cannot estimate platform-wide prevalence or videos per million users. Potentially identifiable account information was not reported.

### 2.2 Eligibility criteria and video classification

Videos were eligible if they were publicly available on Douyin or Bilibili, contained health-related information concerning SGM or LGBTQ+ populations, and provided spoken language, subtitles, or written text sufficient for evaluation. Eligible topics included sexual health, mental health, HIV/STI prevention, PrEP/PEP, transgender and gender-diverse health, healthcare access, stigma, discrimination, or social support.

We excluded duplicates, videos unrelated to SGM health, inaccessible or incomplete videos, and those lacking evaluable health information. Purely entertainment, lifestyle, emotional, or identity-oriented videos without health content were also excluded. Advertisements or commercial promotions were excluded unless they contained substantive health information. Videos containing stigmatizing or discriminatory health claims were retained if they provided evaluable SGM health content, allowing us to assess misinformation and stigma.

Uploader type was categorized as: healthcare professionals; healthcare institutions; public health or community organizations; health science communicators without clearly verified medical qualifications; individual users; commercial accounts; or others. Primary content themes included: general SGM/LGBTQ+ health education; mental health; HIV/STI prevention; PrEP/PEP; sexual health and safer sex; transgender and gender-diverse health; sexual orientation and gender identity; healthcare access and privacy; stigma and social support; human papillomavirus (HPV) vaccination and cancer screening; and other health topics. These categories were based on major SGM health domains and relevant clinical/public health guidance (2, 3, 23–25). For videos covering multiple topics, the primary theme was determined by the dominant message or the topic occupying the largest proportion of the video.

### 2.3 Assessment of quality, reliability, accuracy, and stigmatizing content

We assessed overall video quality using the Global Quality Score (GQS), a 5-point instrument evaluating the quality, usefulness, flow, and educational value of online health information (26). Scores 1–2, 3, and 4–5 indicated low, moderate, and high quality, respectively.

Video reliability was measured with a modified DISCERN instrument, a five-item adaptation of the original DISCERN tool for consumer health information (27, 28). This modified scale assessed five domains: clarity, relevance, traceability, comprehensiveness, and impartiality. Each item scored 0 or 1, yielding a total score from 0 to 5. Scores 0–2, 3, and 4–5 were classified as low, moderate, and high reliability, respectively. Complete GQS and modified DISCERN scoring rules are provided in Supplementary Table S2.

Medical accuracy was evaluated using a study-specific, anchored 0–5 rubric informed by clinical and public health guidance on HIV/STI prevention, PrEP/PEP, safer sexual practices, mental health help-seeking, transgender and gender-diverse health, and healthcare access (24, 29–33). The score reflected the correctness of the central message, consistency with current guidance, adequacy of clinically necessary context, and the presence of unsupported or potentially harmful claims. Misinformation was coded separately for presence and severity. Complete anchors, decision rules, and supporting references are provided in Supplementary Table S3.

Communication framing was assessed with an author-developed exploratory stigma-to-affirmation continuum (0–5), conceptually informed by minority stress theory, destigmatizing digital-design principles, and identity-respecting or affirming care guidance (4, 7, 20, 24). Scores of 0–1 represented explicit or predominant stigmatizing content; 2 represented ambiguous or partially problematic framing; 3 represented neutral, non-stigmatizing communication; and 4–5 represented increasingly active affirmation, including respectful identity language, privacy sensitivity, explicit counter-stigma messaging, and encouragement of evidence-based care. Non-stigmatizing and affirming communication were therefore treated as adjacent but non-equivalent levels: absence of stigma did not by itself constitute active affirmation. The rubric was developed for this study, was not an established or psychometrically validated instrument, and produced one ordinal continuum rather than separate stigma and affirmation domain scores. Complete anchors and interpretive boundaries are provided in Supplementary Table S4.

### 2.4 Evaluation process and statistical analysis

All videos were assessed using a structured consensus-based coding procedure conducted by two trained reviewers with medical or public health backgrounds. Before formal coding, both reviewers received standardized training and jointly evaluated 10 randomly selected videos to calibrate the operational definitions, scoring anchors, and decision rules. During formal coding, the reviewers jointly reviewed each video, discussed the applicable criteria, and assigned a single consensus rating for each variable. Cases for which consensus could not be reached through discussion were referred to a third senior reviewer for adjudication. Because the ratings were generated through consensus rather than through independent parallel assessment, inter-rater agreement statistics were not estimated.

Continuous variables were summarized as medians with interquartile ranges (IQRs), and categorical variables as counts and percentages. Between-platform differences were assessed using the Mann–Whitney *U* test for continuous variables and the chi-square test or Fisher's exact test for categorical variables, as appropriate. For categorical platform comparisons, effect size was summarized using Cramér's *V*; 95% confidence intervals were obtained by inverting the non-central chi-square distribution and transforming the non-centrality limits to the V scale. Differences across uploader types and content themes were examined using the Kruskal–Wallis H test, followed, where appropriate, by Dunn's *post-hoc* comparisons with Bonferroni correction. Associations between cumulative engagement counts and quality, reliability, medical accuracy, misinformation severity, and the exploratory communication score were evaluated using unadjusted Spearman correlations. The unequal final platform sample sizes arose after eligibility screening from equal initial quotas; no significance test of the sample-size difference or user-population weighting was applied because neither addresses selection in a quota-based algorithmic sample. Engagement analyses were descriptive, not adjusted or causal. Analyses were performed using R version 4.3.2 (R Foundation for Statistical Computing, Vienna, Austria); tests were two-sided and *P* < 0.05 denoted statistical significance.

## 3 Results

### 3.1 Video screening and general characteristics

During the prespecified data-collection period, the first 10 results returned for each of 15 search terms were recorded on each platform, yielding 150 candidate records from Douyin and 150 from Bilibili (300 total). After deduplication and eligibility screening, 32 Douyin and 74 Bilibili candidates were excluded, leaving 194 videos (118 Douyin and 76 Bilibili; Figure 1). The platform-specific total exclusions are recoverable from the screening totals; reason-specific exclusion counts were not retained and were not retrospectively reconstructed. Operational details are provided in Supplementary Table S1.

**Figure 1:**
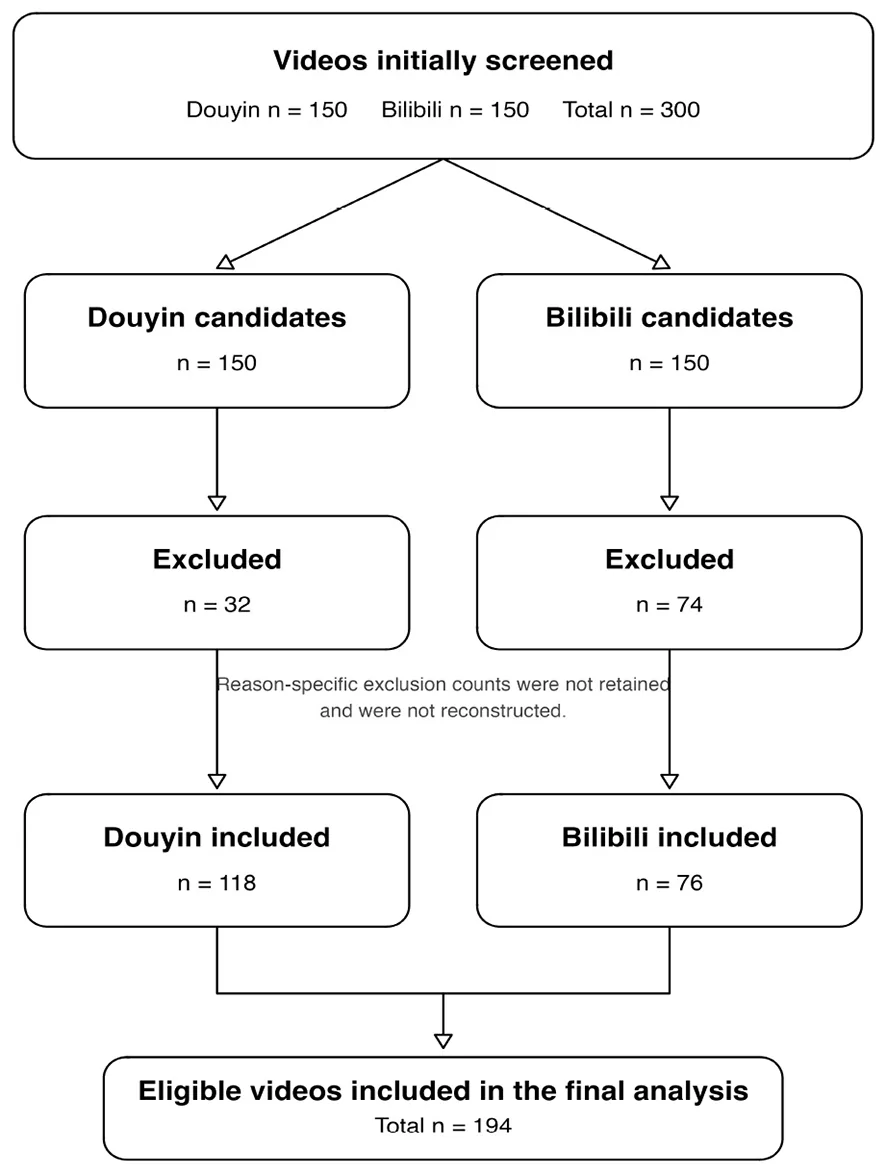
Flowchart of video retrieval, screening, and inclusion. Fifteen search terms were entered separately on each platform, and the first 10 results per term were recorded, yielding 150 candidate records per platform. After deduplication and eligibility screening, 32 Douyin and 74 Bilibili candidates were excluded, leaving 194 eligible videos (Douyin, *n* = 118; Bilibili, *n* = 76). Reason-specific exclusion counts were not retained and were not reconstructed.

Median video duration was 128.5 s (IQR 62.3–231.2). Median likes, comments, saves/collections, and shares were 438.0 (IQR 96.0–1512.0), 32.0 (IQR 8.0–96.0), 186.0 (IQR 41.0–624.0), and 72.0 (IQR 14.0–251.0), respectively. Platform-specific differences were evident: Douyin videos were shorter [median 78.6 s (IQR 45.2–136.8)] but had higher engagement metrics, whereas Bilibili videos were longer [median 196.2 s (IQR 112.2–342.9)], suggesting distinct dissemination patterns.

### 3.2 Uploader types and content theme distribution

Individual users (50/194, 25.8%) and healthcare professionals (49/194, 25.3%) were the most common uploaders, followed by health science communicators without verified credentials (31/194, 16.0%), public health/community organizations (26/194, 13.4%), commercial accounts (17/194, 8.8%), healthcare institutions (17/194, 8.8%), and others (4/194, 2.1%; Figure 2A). Uploader distribution differed by platform (χ^2^ = 21.55, df = 6, *P* = 0.001; Cramér's *V* = 0.33, 95% CI 0.13–0.44). On Douyin, healthcare professionals (35/118, 29.7%) and health science communicators (24/118, 20.3%) were more frequent; on Bilibili, individual users (29/76, 38.2%) and community organizations (16/76, 21.1%) predominated.

**Figure 2:**
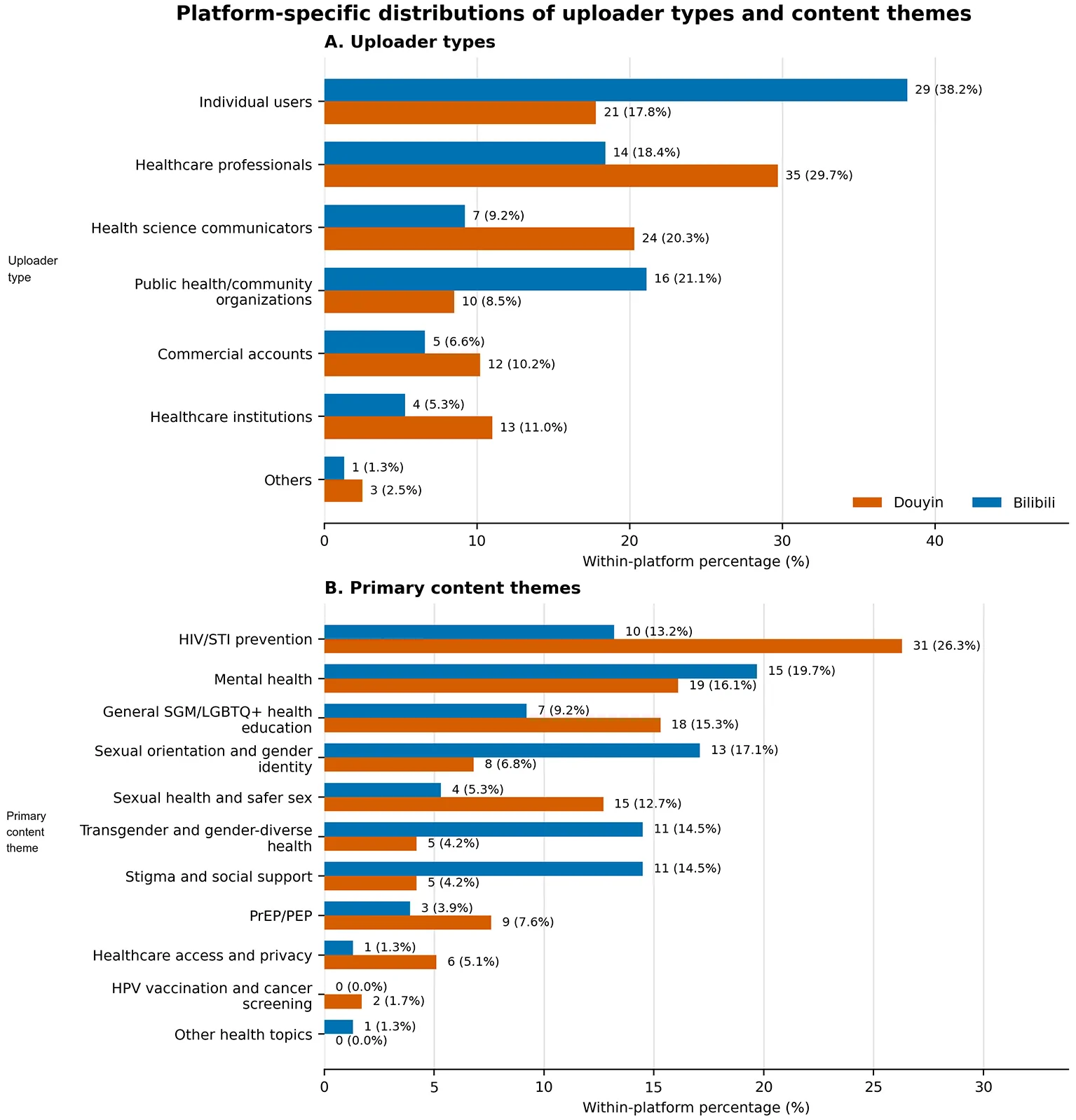
Direct platform comparison of uploader types and primary content themes. Grouped horizontal bars show within-platform percentages for Douyin (*n* = 118) and Bilibili (*n* = 76); labels give n (%). **(A)** presents uploader types and **(B)** presents primary content themes. Categories are ordered for direct visual comparison. SGM, sexual and gender minority; STI, sexually transmitted infection; PrEP, pre-exposure prophylaxis; PEP, post-exposure prophylaxis.

Regarding content themes (Figure 2B), HIV/STI prevention was most common (41/194, 21.1%), followed by mental health (34/194, 17.5%), general SGM health education (25/194, 12.9%), sexual orientation/gender identity (21/194, 10.8%), sexual health/safer sex (19/194, 9.8%), transgender health (16/194, 8.2%), stigma/social support (16/194, 8.2%), PrEP/PEP (12/194, 6.2%), healthcare access/privacy (7/194, 3.6%), HPV vaccination/cancer screening (2/194, 1.0%), and other topics (1/194, 0.5%). Theme distribution differed by platform (χ^2^ = 30.01, df = 10, *P* < 0.001; Cramér's *V* = 0.39, 95% CI 0.16–0.48). Douyin more often featured HIV/STI prevention and sexual health, whereas Bilibili more often featured mental health, sexual orientation/gender identity, and transgender health. Healthcare access and cancer screening were underrepresented on both platforms.

### 3.3 Video quality, reliability, accuracy, and stigmatizing content

Overall quality as measured by the GQS was moderate: median score 3.0 (IQR 2.0–4.0), with the platform-specific distributions of GQS and modified DISCERN categories shown in Figure 3. High-quality videos (GQS 4–5) accounted for 38.1% (74/194). Reliability assessed by modified DISCERN was limited: median score 2.0 (IQR 1.0–3.0), with 19.1% (37/194) classified as highly reliable. Between-platform category differences were not statistically significant for GQS (χ^2^ = 4.70, df = 2, *P* = 0.095; Cramér's *V* = 0.16, 95% CI 0.00–0.28) or modified DISCERN (χ^2^ = 5.21, df = 2, *P* = 0.074; Cramér's *V* = 0.16, 95% CI 0.00–0.29). The medical accuracy score had a median of 3.0 (IQR 2.0–4.0). Misinformation was present in 27.8% (54/194) of videos: minor in 18.6% (36/194), moderate in 7.7% (15/194), and severe in 1.5% (3/194). Common misinformation included incomplete PrEP/PEP explanations, overgeneralized HIV/STI risk claims, and pathologizing descriptions of sexual orientation or gender identity.

**Figure 3:**
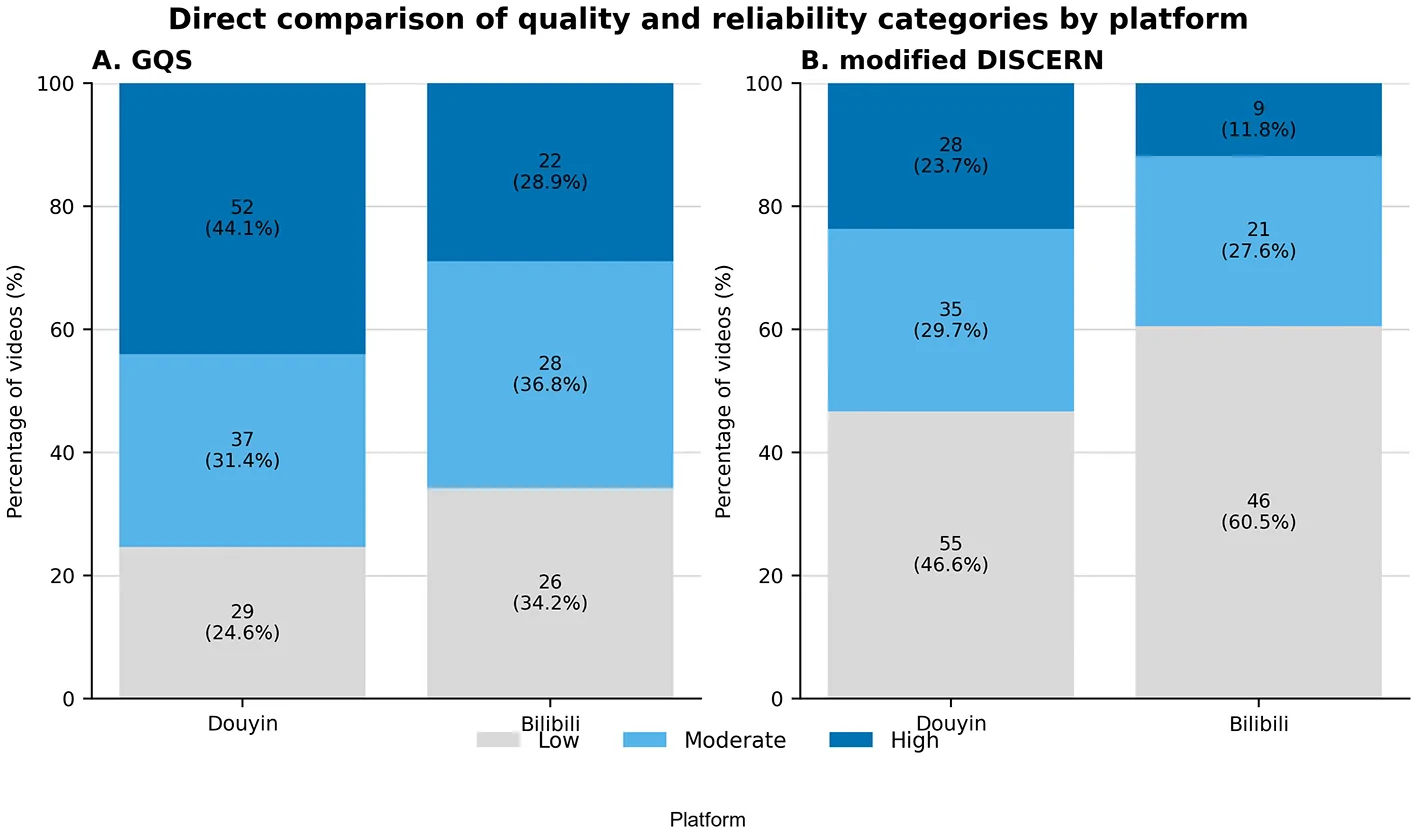
Direct comparison of video quality and reliability categories by platform. One-hundred-percent stacked bars show the proportions of videos classified as low, moderate, or high quality according to the Global Quality Score **(A)** and as low, moderate, or high reliability according to the modified DISCERN score **(B)** for Douyin (*n* = 118) and Bilibili (*n* = 76). Labels give n (%). GQS, Global Quality Score.

The median exploratory stigma-to-affirmation communication score was 3.0 (IQR 2.0–4.0). Overall, 57.2% (111/194) of videos were neutral, non-stigmatizing, or affirming; 25.8% (50/194) had ambiguous or partially problematic framing; and 17.0% (33/194) contained explicit stigmatizing, pathologizing, fear-based, or discriminatory elements. Because this was one ordinal continuum, the combined percentage should not be interpreted as a separate prevalence estimate of active affirmation. The operational definitions and interpretive boundary are provided in Supplementary Table S4.

### 3.4 Differences in video quality across uploader types

Quality and reliability varied significantly by uploader type (Figure 4). Healthcare institutions and healthcare professionals achieved the highest median GQS scores [both 4.0 (IQR 3.0–4.0)], whereas individual users [3.0 (IQR 2.0–3.0)] and commercial accounts [2.0 (IQR 2.0–3.0)] scored lower. Modified DISCERN scores followed a similar pattern, and medical accuracy was higher for professional uploaders. On the exploratory communication continuum, public health/community organizations scored highest, followed by healthcare professionals. Bonferroni-corrected *post-hoc* comparisons showed higher GQS and DISCERN scores for healthcare professionals/institutions than for individual users and commercial accounts, and higher exploratory communication scores for community organizations than for commercial accounts.

**Figure 4:**
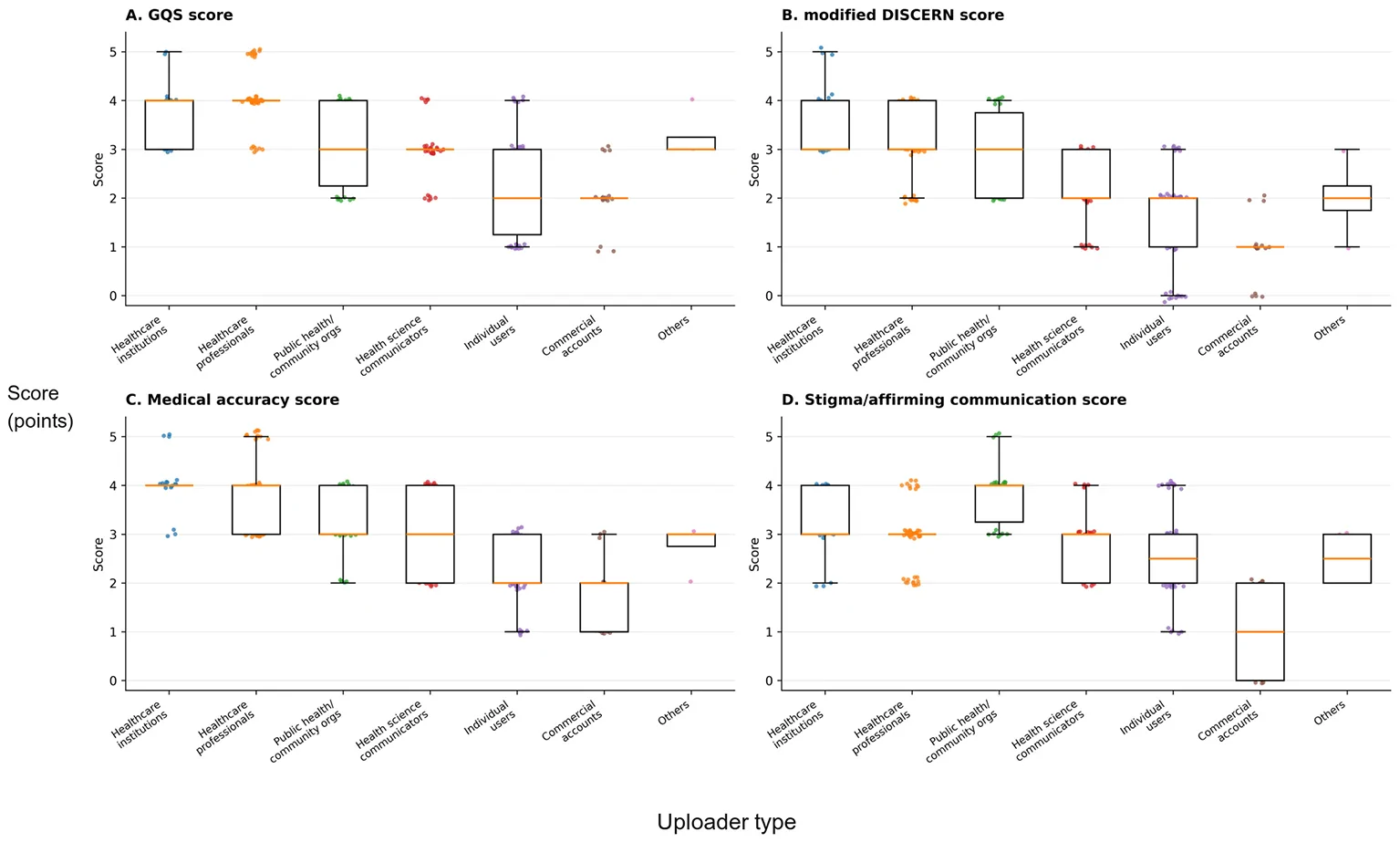
Video quality, reliability, medical accuracy, and exploratory stigma-to-affirmation communication scores by uploader type. Boxplots with jittered points show differences across uploader types. **(A–D)** show GQS, modified DISCERN, medical accuracy, and the exploratory communication score, respectively. Boxes indicate interquartile ranges, horizontal lines indicate medians, whiskers indicate ranges excluding outliers, and dots represent videos. Higher communication scores indicate movement from more stigmatizing toward more affirming framing; the score is a single author-developed ordinal continuum, not two independently validated domains. SGM, sexual and gender minority.

### 3.5 Association between user engagement and video quality

Unadjusted Spearman analysis (Figure 5) showed strong positive intercorrelations among cumulative engagement metrics: likes correlated with saves/collections (*r* = 0.80), comments (*r* = 0.76), and shares (*r* = 0.73); saves/collections correlated with shares (*r* = 0.84); and comments correlated with saves/collections (*r* = 0.71) and shares (*r* = 0.69). In contrast, raw engagement counts showed weak or negligible correlations with quality-related indicators: likes vs. GQS (*r* = 0.10), modified DISCERN (*r* = 0.06), medical accuracy (*r* = 0.08), and the exploratory communication score (*r* = −0.04). Video duration showed weak positive correlations with GQS (*r* = 0.22) and modified DISCERN (*r* = 0.19). GQS correlated with modified DISCERN (*r* = 0.56), medical accuracy (*r* = 0.38), and the exploratory communication score (*r* = 0.31). Medical accuracy correlated negatively with misinformation severity (*r* = −0.49), which also correlated negatively with the exploratory communication score (*r* = −0.43). These are unadjusted descriptive relationships in the selected sample.

**Figure 5:**
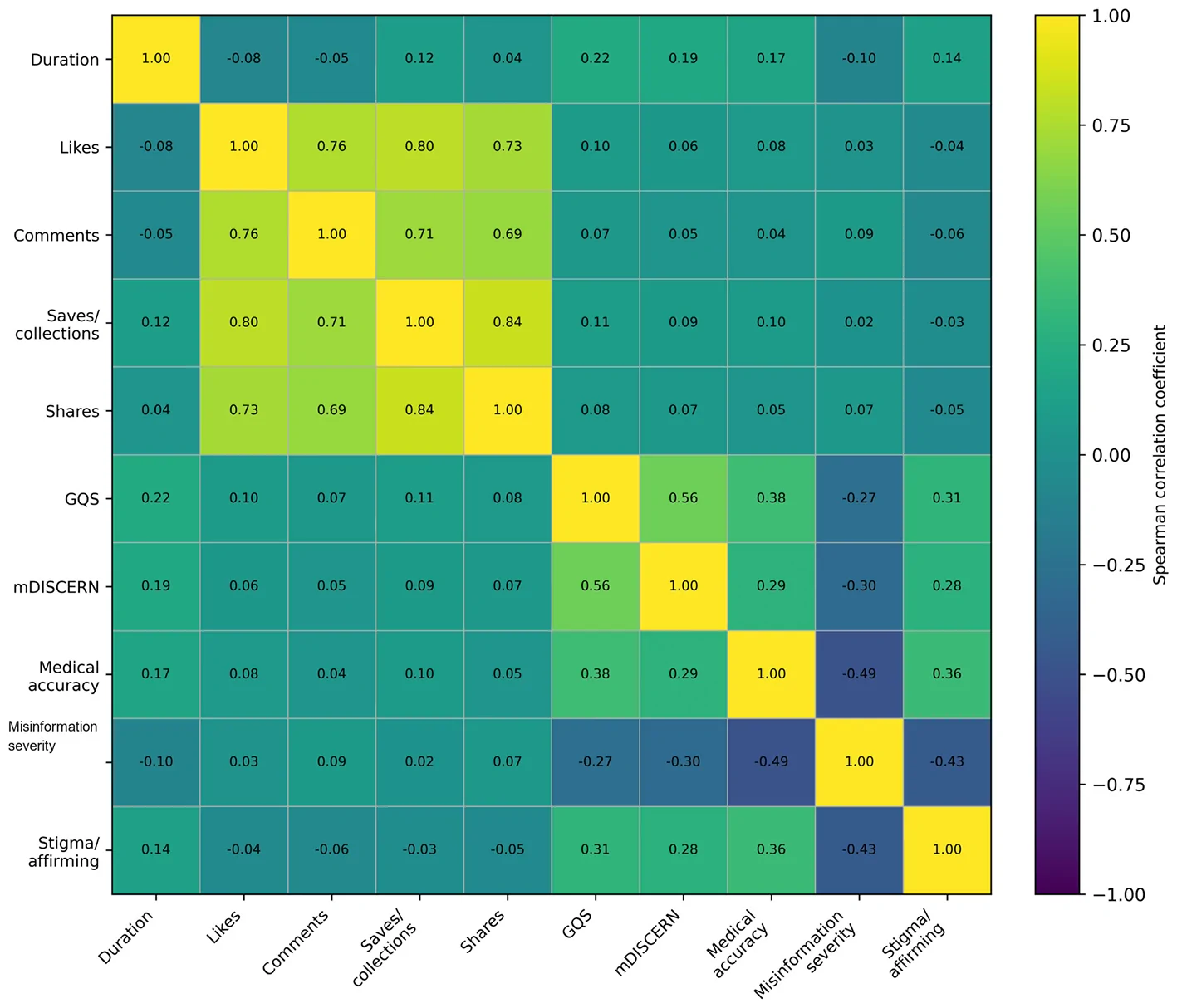
Spearman correlations between engagement metrics and video quality indicators. The heatmap shows coefficients among cumulative engagement metrics, video duration, GQS, modified DISCERN, medical accuracy, misinformation severity, and the exploratory stigma-to-affirmation communication score. Engagement metrics were strongly intercorrelated but showed weak or negligible correlations with quality-related indicators. Higher misinformation severity was negatively correlated with medical accuracy and the exploratory communication score. GQS, Global Quality Score; SGM, sexual and gender minority.

## 4 Discussion

In this cross-sectional analysis of 194 prominently ranked SGM health-related videos, we identified substantial heterogeneity in content sources, themes, quality, reliability, and communication framing. HIV/STI prevention and mental health predominated, whereas healthcare access, privacy protection, HPV vaccination, and cancer screening were underrepresented. Overall quality was moderate, source transparency and reliability were limited, more than one-quarter of videos contained misinformation, and 17.0% included explicitly stigmatizing, pathologizing, fear-based, or discriminatory elements. Engagement metrics were strongly intercorrelated but weakly associated with quality-related measures. These findings describe content surfaced by a predefined, time-bounded search rather than platform-wide prevalence.

The platforms exhibited distinct uploader and content profiles, with moderate platform associations for uploader type and theme. These differences may reflect platform-specific formats, creator communities, audiences, and ranking systems as well as the unequal final sample sizes. Videos from healthcare professionals and institutions generally had higher quality, reliability, and medical accuracy scores, whereas community organizations scored comparatively well on the exploratory communication continuum. Medical expertise and community-informed communication may therefore offer complementary strengths, but the observed platform differences should not be interpreted as independent platform effects.

The distribution of content themes also revealed clinically relevant gaps. Although HIV/STI prevention and mental health represent important health priorities, their predominance may inadvertently reinforce an overly narrow representation of SGM health as being defined primarily by infection risk or psychological distress. Preventive care, cancer screening, healthcare navigation, privacy protection, reproductive health, and healthy aging received substantially less attention. Comprehensive SGM healthcare extends beyond disease prevention to encompass equitable access, culturally competent clinical services, preventive care across the life course, and protection from discrimination (2, 3, 24). Broadening the thematic scope of online content may therefore help counter stereotyped representations of SGM health and better address the diverse healthcare needs of these populations.

Information reliability was a particular concern. Many videos did not clearly identify their sources, cite supporting evidence, or acknowledge clinical uncertainty and limitations. Misinformation was identified in 27.8% of the included videos and most commonly involved HIV/STI risk, PrEP/PEP, sexual health, or pathologizing interpretations of sexual orientation and gender identity. Incomplete or oversimplified explanations of PrEP and PEP may be especially consequential because their appropriate use depends on timing, adherence, eligibility assessment, follow-up testing, and professional consultation. Individuals who encounter stigma or barriers to in-person healthcare may rely disproportionately on online sources, potentially increasing the influence of inaccurate or incomplete information (3, 5, 10, 11, 14). Research on short-video misinformation and medical advice from social media influencers also underscores the challenges users face when evaluating health claims and making informed decisions (34, 35). Platforms and content creators should therefore prioritize transparent source attribution, links to authoritative guidance, and clear communication of uncertainty.

Minority stress theory helps interpret why communication framing matters beyond factual accuracy. Stigmatizing, discriminatory, pathologizing, and fear-based messages can function as distal minority-stress exposures, while repeated exposure may contribute to proximal processes such as expectations of rejection or internalized stigma (4, 7, 36). Conversely, identity-respecting, privacy-sensitive, counter-stigma communication may reduce harmful framing, although the present content analysis did not measure viewer responses. The author-developed score placed neutral non-stigmatizing communication and active affirmation at different points on one exploratory continuum; it did not establish separate validated domains. Accordingly, these findings support attention to communication framing but do not demonstrate causal effects on stress, trust, or healthcare seeking.

Cumulative engagement metrics showed only weak unadjusted associations with video quality, reliability, medical accuracy, and the exploratory communication score. Likes, comments, saves, and shares should not be interpreted as direct indicators of evidentiary value. Engagement reflects time since publication, audience exposure, uploader reach, follower numbers, format, emotional salience, and platform-specific recommendation processes. Because these factors were not fully measured or controlled, the observed correlations are descriptive rather than evidence of an independent relationship between popularity and content quality. Similar platform- and creator-related heterogeneity in the quality of short-video health information has also been reported in studies of neonatal jaundice and diabetic retinopathy ((35, 36)).

These findings have implications for content creators, healthcare organizations, community groups, and platforms. Healthcare professionals and institutions could improve source transparency, acknowledge uncertainty, use accessible language, and adopt identity-respecting and privacy-sensitive framing. Community organizations could strengthen clinical reliability through collaboration with qualified healthcare professionals. Platforms could facilitate access to authoritative resources, improve review mechanisms for harmful misinformation, and promote underrepresented dimensions of SGM health. Such approaches may improve the accuracy, inclusiveness, and clinical usefulness of the content users encounter.

Several limitations should be considered. First, the 15 search terms were research-team-developed formal terms and were not validated against focus groups, preliminary user surveys, platform search logs, or a user-facing pilot. Colloquial or euphemistic queries may therefore have surfaced additional content. Second, selecting the first 10 default-ranked results per term produced a non-probability, time-specific algorithmic snapshot, not an exhaustive sample. Rankings can change with proprietary algorithms and temporal trends, and Douyin and Bilibili have materially different recommendation, community, and subscription ecologies. Equal initial quotas standardized retrieval but did not make the final samples representative of either platform. Differential exclusions produced 118 Douyin and 76 Bilibili videos, which can affect precision and power. Weighting by platform user population or reporting videos per million users would not correct this selection process because the design supplied neither a probability sample nor a valid platform-level denominator.

Third, the medical accuracy rubric and exploratory stigma-to-affirmation continuum were developed for this study and have not undergone external psychometric validation. Non-stigmatizing and actively affirming communication are conceptually distinct, but the original protocol recorded a single ordinal continuum rather than separate item-level stigma and affirmation domain scores. Retrospective separation was not feasible because the required item-level source ratings were not retained and some bookmarked videos had subsequently been removed, made private, or otherwise become inaccessible. We did not impute or reconstruct unavailable ratings. Consensus rather than independent parallel coding also precluded inter-rater agreement estimates. Future prospective studies should archive permissible metadata, preserve item-level independent ratings, validate separate stigma and affirmation domains, and report agreement. Fourth, cumulative engagement measures reflected unequal exposure and unmeasured promotion. Finally, this study assessed video content, not viewer knowledge, proximal minority-stress processes, healthcare decisions, or health outcomes.

Overall, prominently ranked SGM health videos in this time-bounded sample showed moderate quality, limited reliability, uneven thematic coverage, and persistent misinformation and stigmatizing framing. Professional uploaders generally provided more accurate and reliable information, while community organizations scored comparatively well on the exploratory communication continuum. Cumulative engagement did not provide a reliable proxy for content quality. These findings should not be generalized to all SGM health content on either platform. Prospective studies with user-informed search terms, reproducible time-stamped sampling, independently coded stigma and affirmation domains, and preserved item-level metadata are needed.

## Data Availability

The data supporting the conclusions of this article will be made available by the corresponding author (yangfei@301hospital.com.cn), upon reasonable request.
