# Supplementary material for: Quality, reliability, and stigmatizing content of sexual and gender minority health videos on Chinese short-form video platforms
Source: Front Public Health. 2026 Oct 14;14:1915092. doi: 10.3389/fpubh.2026.1915092 (PMC13713801; doi:10.3389/fpubh.2026.1915092)
Supplement: Supplementary file 1 [file Table_1.docx]

### Supplementary Table S1. Complete search and sampling protocol

| **Element** | **Operational detail** |
| --- | --- |
| Data-collection period | January 1–May 1, 2026 |
| Account conditions | New accounts with no prior search, viewing, engagement, or following history |
| Search mode | Platform-default comprehensive/relevance ranking. |
| Queries | 15 terms (English gloss [Chinese original]): LGBT health [LGBT健康]; LGBTQ health [LGBTQ健康]; sexual-minority health [性少数健康]; sexual-minority mental health [性少数心理健康]; gay and lesbian health [同性恋健康]; transgender health [跨性别健康]; gender identity [性别认同]; sexual orientation [性取向]; HIV prevention [HIV预防]; AIDS prevention [艾滋病预防]; STI prevention [性病预防]; pre-exposure prophylaxis [暴露前预防]; post-exposure prophylaxis [暴露后预防]; PrEP; PEP. |
| Records per query | First 10 returned videos per search term. |
| Initial candidate records | 150 per platform; 300 total before deduplication |
| Combining results | Pooled separately within each platform; all triggering terms and highest observed rank retained |
| Within-platform duplicates | Same platform-specific video ID or URL; retained once |
| Cross-platform reposts | Treated as platform-specific records because engagement metrics were platform-specific. |
| Exclusion hierarchy | Duplicate → unrelated/irrelevant → inaccessible or incomplete → no evaluable health information. |
| Rationale for 150/platform | A priori balance of coverage across 15 queries and the feasibility of structured consensus-based coding; not intended as a probability sample |
| Search-term provenance | Developed a priori by the research team from formal terminology in clinical/public health guidance and prior SGM health literature; no focus groups, user logs, or formal user-facing pretest. |
| Search-term limitation | Formal terms may miss colloquial or euphemistic searches, including terms analogous to “circle,” “rainbow,” or “gay.” |
| Ranking snapshot | The top 10 default-ranked results captured a time-specific algorithmic snapshot; rankings were dynamic, platform-specific, and neither exhaustive nor representative. |
| Platform-specific screening | Screened: Douyin n = 150 and Bilibili n = 150 (total n = 300). Excluded after deduplication/eligibility screening: Douyin n = 32 and Bilibili n = 74. Included: Douyin n = 118 and Bilibili n = 76 (total n = 194). |
| Reason-specific exclusions | Reason-specific counts were not retained and were not retrospectively reconstructed. |
| Archiving limitation | Raw videos were not downloaded or redistributed; some bookmarked videos subsequently became unavailable. |

### Supplementary Table S2. GQS and modified DISCERN scoring rules

| **Score/item** | **Operational definition** |
| --- | --- |
| GQS 1 | Poor quality, poor flow, most information missing, and not useful for health education |
| GQS 2 | Generally poor quality and flow; some information present but important topics missing; limited usefulness |
| GQS 3 | Moderate quality; some important information adequately discussed, but other clinically relevant information is incomplete |
| GQS 4 | Good quality and generally useful; most relevant information addressed with only minor omissions |
| GQS 5 | Excellent quality, flow, and usefulness; comprehensive, clear, and highly useful for health education |
| Modified DISCERN item 1: clarity | 1 if the purpose and central message are clear and understandable; otherwise 0 |
| Modified DISCERN item 2: relevance | 1 if the content directly addresses the stated health topic and intended audience; otherwise 0 |
| Modified DISCERN item 3: traceability | 1 if clinical claims are linked to identifiable professional expertise, guidelines, references, or other verifiable sources; otherwise 0 |
| Modified DISCERN item 4: comprehensiveness | 1 if clinically important benefits, risks, alternatives, limitations, or uncertainty are addressed when relevant; otherwise 0 |
| Modified DISCERN item 5: impartiality | 1 if the presentation is balanced and avoids unsupported promotion, exaggeration, or one-sided claims; otherwise 0 |
| Modified DISCERN total | Sum of five binary items (0–5); 0–2 low, 3 moderate, and 4–5 high reliability. |

### Supplementary Table S3. Medical accuracy and misinformation rubric

| **Score/rule** | **Operational definition** |
| --- | --- |
| Medical accuracy 5 | All clinically relevant claims are consistent with current evidence/guidance; necessary context is provided; no material error or omission |
| Medical accuracy 4 | Generally accurate; at most one minor imprecision or omission unlikely to affect health decisions |
| Medical accuracy 3 | Main message is evidence-consistent, but limited imprecision or omission could cause some misunderstanding |
| Medical accuracy 2 | Mixed accuracy; at least one substantive error or omission could influence health understanding or decisions |
| Medical accuracy 1 | Predominantly inaccurate or misleading, with only limited accurate information |
| Medical accuracy 0 | Entirely or almost entirely inaccurate, promotes a potentially harmful action, or actively discourages indicated evidence-based care |
| Misinformation 0: none | No claim contradicting the prespecified medical reference standards |
| Misinformation 1: minor | Localized imprecision or omission unlikely to materially change health behavior or cause harm |
| Misinformation 2: moderate | Error or omission could lead to meaningful misunderstanding, inappropriate self-management, delayed care, or moderate psychosocial harm |
| Misinformation 3: severe | Claim is likely to cause serious physical or psychosocial harm, promotes a dangerous intervention, or discourages urgent/indicated care |
| Multiple errors | Assign the severity of the most consequential inaccurate claim; record each error domain separately in the coding form |
| Reference standards | WPATH Standards of Care Version 8 [24]; WHO consolidated HIV guidelines (2021) [29]; CDC Sexually Transmitted Infections Treatment Guidelines (2021) [30]; CDC Clinical Guidance for PrEP (updated 2026) [31]; CDC Clinical Guidance for PEP (2025) [32]; and WHO mhGAP Intervention Guide Version 2.0 (2016) [33]. |

### Supplementary Table S4. Exploratory stigma-to-affirmation communication rubric

| **Score** | **Operational definition** |
| --- | --- |
| 0 | Explicitly pathologizing, discriminatory, hostile, or dehumanizing communication about SGM identities |
| 1 | Predominantly stigmatizing communication, including shame-, fear-, or morality-based framing, with little neutral or affirming context |
| 2 | Ambiguous or partially problematic framing, such as an unsupported identity–disease association, stereotype, or insensitive wording, without explicit hostility |
| 3 | Neutral and non-stigmatizing communication; identities are described respectfully, but affirming or privacy-sensitive guidance is limited |
| 4 | Affirming and inclusive communication that respects diverse identities and encourages appropriate evidence-based help-seeking |
| 5 | Strongly affirming, culturally responsive, privacy-sensitive, and balanced communication that explicitly counters stigma or supports equitable access to care |
| Coding domains considered | Pathologizing language; identity–disease/risk stereotyping; shame/fear/moralizing framing; discriminatory terms; respect for identities; privacy protection; evidence-based consultation |
| Overall categories | Scores 0–1: explicit/predominant stigmatizing content; score 2: ambiguous/partially problematic; score 3: neutral/non-stigmatizing; scores 4–5: affirming |
| Construct status | Author-developed exploratory ordinal continuum; not an established or psychometrically validated standard instrument. |
| Conceptual basis | Minority stress theory, destigmatizing digital-design principles, and identity-respecting/affirming care guidance [4,7,20,24]. |
| Interpretive boundary | Non-stigmatizing and affirming communication are not interchangeable. Score 3 denotes neutral/non-stigmatizing communication; scores 4–5 require increasingly active affirmation. Absence of stigma alone does not constitute affirmation. |
| Domain structure | One overall ordinal continuum was recorded. Separate item-level stigma and affirmation domain scores were not retained and should not be inferred from the total. |
| Retrospective limitation | Separate re-coding was not performed because the required item-level source ratings were unavailable and some videos had been removed, made private, or become inaccessible. |
